# The illusion of righteousness: corporate social responsibility practices of the alcohol industry

**DOI:** 10.1186/1471-2458-13-630

**Published:** 2013-07-03

**Authors:** Sungwon Yoon, Tai-Hing Lam

**Affiliations:** 1 Department of Community Medicine & School of Public Health, Li Ka Shing Faculty of Medicine, The University of Hong Kong, Hong Kong, SAR, China

**Keywords:** Alcohol industry, Corporate social responsibility, Politics, Alcohol policies, Framework convention

## Abstract

**Background:**

Corporate social responsibility (CSR) has become an integral element of how the alcohol industry promotes itself. The existing analyses of CSR in the alcohol industry point to the misleading nature of these CSR practices. Yet, research has been relatively sparse on how the alcohol industry advances CSR in an attempt to facilitate underlying business interests, and in what ways the ongoing display of industry CSR impacts public health. This paper aims to investigate the alcohol industry’s recent CSR engagements and explain how CSR forms part of the industry’s wider political and corporate strategies.

**Methods:**

Our study used qualitative methods to collect and analyse data. We searched for materials pertaining to CSR activities from websites of three transnational alcohol corporations, social media platforms, media reports and other sources. Relevant documents were thematically analysed with an iterative approach.

**Results:**

Our analysis identified three CSR tactics employed by the alcohol companies which are closely tied in with the industry’s underlying corporate intents. First, the alcohol manufacturers employ CSR as a means to frame issues, define problems and guide policy debates. In doing this, the alcohol companies are able to deflect and shift the blame from those who manufacture and promote alcoholic products to those who consume them. Second, the alcohol corporations promote CSR initiatives on voluntary regulation in order to delay and offset alcohol control legislation. Third, the alcohol corporations undertake philanthropic sponsorships as a means of indirect brand marketing as well as gaining preferential access to emerging alcohol markets.

**Conclusions:**

The increasing penetration and involvement of the alcohol industry into CSR highlights the urgent needs for public health counter actions. Implementation of any alcohol control measures should include banning or restricting the publicity efforts of the industry’s CSR and informing the public of the alcohol industry’s notion of social responsibility. More significantly, an internationally binding instrument should be called for to enable countries to differentiate between genuine concerns and spurious altruism, and in doing so, resist the industry’s attempt to erode alcohol control.

## Background

Corporate social responsibility (CSR) has become an integral element of the alcohol industry’s self-representation and image. With renewed public awareness of the serious harm caused by alcohol consumption and the prospect of adverse implications on profits, a growing number of alcohol corporations are competing with each other to adopt CSR strategies in an attempt to portray themselves as good corporate citizens. Major transnational alcohol manufactures have websites dedicated to CSR which display various CSR related programmes and/or campaigns. These invariably include or involve some sort of sponsorship schemes, public awareness talks or dialogues, education programmes, networking events, and partnerships with government as well as voluntary codes of practice for marketing and advertising. They openly profile themselves as socially responsible actors who are willing to embrace societal concerns on alcohol-related harm.

In its original sense, the term CSR is defined as a moral and stakeholder obligation [1], emanating from a notion that business is responsible to society in general and thus corporations should be answerable to those who directly or indirectly affect or are affected by a firm’s activity [2]. Whereas the overall value of the alcohol industry’s CSR remains questionable, public health advocates note the fundamental contradictions between the alcohol industry’s claims of responsibility and their continuing promotion of alcoholic products.

For instance, Hill views the alcohol industry’s CSR as a public relations strategy which may serve to promote the global marketing of alcohol rather than addressing the health impact and risks inherent in alcoholic products [3]. Similarly, Casswell [4] and others [5-7] contend that the primary role of industry-supported social aspect organizations is not to enhance public health but to influence decision makers and government policy while promoting ineffective interventions. The alcohol industry’s conflict of interest is so marked that today a growing body of literature takes the view that the alcohol industry takes advantage of CSR rhetoric in an attempt to achieve corporate interests [8-11]. Such literature also suggests that the alcohol industry’s CSR engagement is not only a mechanism for the preservation of corporate interests but a platform through which members of the industry seek to invalidate a broader public health perspective on problems associated with alcohol consumption and influence the public and policy makers [12]. The alcohol industry’s CSR activities are, in the words of Peter Anderson, “a communication device to delay policy – and, they work [13].”

Despite an abundance of widely available literature pointing to the existence of inconsistencies between the alcohol industry’s CSR claims and how they conduct their business in practice, there is a general paucity of critical examination of the alcohol industry’s CSR activities. Existing analyses of CSR initiatives tend to focus on the likelihood of discrepancy between the rhetoric and actual practices of alcohol corporations. Yet, when studies shift their focus to expose the industry’s hidden agendas behind their CSR activities, such studies are inclined to “acknowledge” the industry’s motivations to develop CSR initiatives rather than investigating the details of how the industry employed CSR to advance their interest in practice [14,15] Furthermore, whilst much of the literature on the alcohol industry recognises that CSR functions as part of the industry’s public relations strategy, relatively little has been done to critically examine the political nature of the alcohol industry’s CSR activities [16,17].

Drawing on an analysis of the global alcohol industry’s documents and other relevant materials, this paper aims to investigate the alcohol industry’s recent CSR engagements and explain how CSR forms part of the industry’s wider political and corporate strategies. This paper particularly focuses on examining precisely what the alcohol industry aspires to gain from CSR, how they advance their CSR in an attempt to facilitate this underlying intent, and to what extent the ongoing display of industry CSR poses a potentially significant challenge to public health and effective alcohol control. Our analysis addresses the nature and impact of the alcohol industry’s intensifying CSR activities on a global scale to highlight the need for collective global action and regulatory framework to safeguard public health in response to the behaviour and practices of transnational alcohol corporations.

## Methods

Our study used qualitative methods to collect and analyse data. We searched for materials from three major alcohol manufacturers in the market: Pernod Ricard, SABMiller, and Anheuser-Busch InBev (AB InBev). These alcohol manufacturers were selected on the basis of their market dominance in the global sale of alcoholic beverages [18,19]. In addition to alcohol companies, the International Centre for Alcohol Policies (ICAP), one of the alcohol industry’s social aspect organizations, was included in our data collection since such organizations represent the industry’s CSR views and perspectives.

Data search began with the websites of the three alcohol manufacturers and ICAP since the alcohol industry tactically utilizes company websites to exercise public relations and publicize their activities. Key terms such as “corporate social responsibility,” “responsible drinking,” “ethics,” “sustainable development,” “philanthropy,” “corporate citizenship,” and “corporate code of conduct” were used and relevant terms were identified by the snowball technique. This yielded commentaries, policy statements, speeches, annual reports, press releases, newsletters, interviews, annual summaries of CSR activities, presentations, and transcripts of meetings. Although searches were not confined to a specific time frame, materials dated between 2008 and 2012 provided most of the information.

Additional materials such as online pamphlets and outlines of event planning were obtained through email correspondence with the APCO Worldwide, a public relations agency for the alcohol industry that contacted us to solicit participation (which we declined) in one of the alcohol manufacturer’s local public event on responsible drinking. Facebook pages and Youtube channels for the alcohol companies listed above were accessed to identify CSR messages placed on these two social media platforms. We also searched LexisNexis, Newsbank and internet search engines using the same search terms between December 2011 and January 2013 for relevant coverage and contextualization.

A total of 368 industry materials and 578 media reports were collected. The materials were screened to include only those that primarily concerned industry tactics and arguments on CSR. Therefore materials pertaining to the institutional actions, programmes and campaigns, and strategic plans aimed at publicizing CSR engagements were included for analysis. We excluded materials according to the following criteria: (1) documents that are written in languages other than English; (2) materials that focus on corporate activities but considered unrelated to the CSR (e.g. market trends, product sales, management structure); (3) materials where CSR claims and preferences of a particular corporate entity are not articulated; and (4) duplicates (e.g. draft and final forms, PDF and interactive versions). These criteria were met by 281 documents.

These materials were imported into NVivo 10 and a thematic analysis based upon grounded theory was conducted [20]. In the present study, coding categories were developed progressing from open coding to analytical coding in a cyclical process to ensure that analysis involved continual interaction with the data. Special efforts were made to ensure that the coding represents consensus in how the alcohol industry characterized CSR initiatives. While each item was coded by the lead author, the second author took part in the data coding process to provide an indication of the accuracy of theme generation and allocation. This involved the coding of a proportion of the materials to enable the coding frame to be clarified and modified. Results of the coding frame were shared and areas of discrepancy were discussed to ensure that the findings reflect an accurate interpretation of the industry rhetoric and practice.

## Results

### CSR as an instrument for framing alcohol-related problems: personal responsibility

The three alcohol corporations appeared to have devoted considerable resources to the framing of issues on alcohol consumption as part of their CSR initiatives. Some companies set up front groups or use PR companies alongside a number of other communications platforms to disseminate and propagate their commitments to what is called “responsible drinking”. For instance, in early 2012, Pernod Ricard chose APCO Worldwide to develop the implementation plan for the project on responsible drinking habits [21]. APCO Worldwide is a public relations company that “helps clients anticipate what's next and smartly manage reputational, communication and business opportunities and challenges that affect their organizations, products, services or brands [22].” It is well known that APCO Worldwide has worked for companies in the tobacco industry, including *British American Tobacco, Philip Morris* and *Brown and Williamson* on issues related to the industry’s sponsorship of CSR [23]. On 17 January 2012, on behalf of Pernod Ricard Asia, APCO Worldwide drafted a proposal on the implementation of public events on “Alcohol & Youth” with an effort to significantly “raise awareness on CSR and responsible drinking” and to heighten “external visibility [24].” Likewise, SABMiller created a dedicated website called *Talking Alcohol*[25] in close collaboration with *Drinkware Trust*[26], a UK-based social aspect organization sponsored by the industry. The site provides views on alcohol in general as well as information about alcohol consumption.

At the centre of the responsible drinking initiative is the promotion of a core idea built on the alcohol industry’s corporate interest: the value of personal responsibility [27]. It is important to note that the nature and causes of alcohol-related problems can be framed in widely different ways which can subsequently inform different response measures. In other words, a problem framed as a matter of personal responsibility can be addressed differently from one that is dues to other factors such as corporate misconduct or a social environment in which alcohol is consumed. Alcohol companies promote the view that alcohol confers benefits and pleasures, and that it should be thought of primarily as an aid to recreation and possibly as beneficial to health. For example, SABMiller’s CSR website states that “our beer adds to the enjoyment of life for the overwhelming majority of consumers….alcohol may **provide physical benefits** for some people when consumed in moderation [28].” [emphasis added] It typically follows an assertion that alcohol manufacturers simply provide choices and pleasure, and do not promote the abuse of their product. The idea underpinning this argument is that reckless drinking of individual consumers is the root cause of alcohol-related problems. SABMiller’s CSR website suggests that:

*Drinking alcohol is a****matter of individual judgment and accountability****. It’s been a part of social life and celebrations around the world for thousands of years. Drinking sensibly means you can enjoy yourself – and stay safe*[29]*.* [emphasis added]

Accordingly, the alcohol industry’s CSR rhetoric conveys the idea that maintaining a sense of individual responsibility is the key to preventing alcohol-related harms and therefore excessive or inappropriate consumption among certain individuals are to be blamed and controlled. For example, in its CSR document, Pernod Ricard states that:

*Alcohol is enjoyed by many people around the world because of its relaxing properties, as an enhancer of sociability and as a complement to meals. Alcohol can also****create problems for individuals who drink it irresponsibly and in exces****s*[30]. [emphasis added]

Alcohol companies in general acknowledge the social harms associated with alcohol consumption such as crime, violence, homicide and drunk driving. But nevertheless, the industry’s rhetoric deliberately avoids attention to corporate sources of alcohol problems by targeting individual drinkers as deviant for harming others and violating rules. SABMiller comments in its position statements that:

***We acknowledge that while alcohol does not cause violence****, some people who commit acts of violence might have also consumed alcohol. The relationship between alcohol consumption and violent behaviour is however extremely complex ….****those who choose to drink too much, to be violent, or both must be held fully accountable for their choices and actions***[31]*.* [emphasis added]

Note that the rhetoric on the private moral choices of drinkers and individual responsibility enables alcohol corporations to be selective about which areas of alcohol policies are to be adopted or eliminated. First of all, alcohol companies do not support the kind of across-the-board regulation, particularly those that seek to control overall alcohol consumption levels because such measures pose a serious threat to the industry’s profits. Instead, the industry employs a dissecting narrative which cuts across and targets different segments of the population. Alcohol companies contend that population-based measures alone are inadequate in tackling alcohol-related problems. On its CSR website, ICAP asserts that:

*It [alcohol industry’s CSR] begins with the recognition that****one-size-fits-all solutions are generally not effective****. We [ICAP] have consistently seen evidence that such approaches are unrealistic and that the initiatives most effective in preventing and reducing alcohol-related harms are those****tailored to regional and local societies and cultures***[32]*.* [emphasis added]

SABMiller shares the view that “Different things work in different markets. So our efforts [on alcohol responsibility] are locally designed and run with help from local partners [33].” In essence, the industry narratives indicate that they do not support any measures that curb overall demand (and presumed consumption) through restrictions.

Second, since the alcohol industry claimed that problems are usually caused by misusers, namely a “higher risk group”, one should educate consumers on drinking responsibly. Alcohol companies are a strong proponent of alcohol education and campaigns which are portrayed as the most effective way of tackling alcohol-related harm. All three companies in this study have actively engaged in a variety of education programmes as part of their CSR strategies. For instance, AB InBev claims to have invested more than USD $300 million in awareness and education programmes for responsible drinking [34]. Pernod Ricard announced that they have financed more than 50 projects on responsible drinking awareness as stated on its CSR website [35]. Likewise, SABMiller claims to have invested USD $875 million toward the promotion of responsible drinking programmes [36]. SABMiller stated that:

*Our efforts will****continue to focus on education and awareness initiatives****, especially in developing countries, with a particular emphasis on at-risk young people and those affected by the harmful drinking of others, as emphasized by the WHO strategy*[37]*.* [emphasis added]

The alcohol companies’ CSR initiatives on the education of problem drinkers and young drinkers do not represent the whole story of the industry’s CSR tactics. More recent CSR initiatives show that the industry has attempted to highlight the role of parents as an agent responsible for young people’s drinking practices and access to alcohol. For example, Pernod Ricard launched parent education and awareness campaigns as part of the company’s underage drinking prevention programme. It claims that “Latest studies helped to shed light on the formative influence of parental attitude on the drinking habits of minors. The more a child feels permitted to drink, the more he or she risks developing an alcohol dependency [38].” The emphasis on parents in alcohol education of children tends to be consistent across alcohol companies. SABMiller implemented the *Family Talk About Drinking* programme aiming to encourage communication between parents and children [39]. While strong parental ties may be crucial to encouraging children to make appropriate decisions about alcohol, there is a risk that such an emphasis can fundamentally shift the focus of responsibility from the industry’s corporate practice in aggressive alcohol marketing and promotion to parents (or adults) who raise young children.

The alcohol companies’ documents also illustrate that the alcohol industry’s CSR on education and public awareness tends to shift the provision of educational information from traditional school-based education and public campaigns to web-based sources and other communication channels such as mobile devices and social media with unprecedented sophistication, an approach regarded by public health experts as a powerful marketing tool for the industry to target youth and promote alcohol brands [40]. For example, AB InBev created the *Family Talk About Drinking Program* on Facebook with 54,921 people liking the page as of December 2012 [41]. SABMiller posted a wide range of video clips on responsible drinking and other related CSR activities on YouTube [42]. Likewise, Pernod Ricard launched a Facebook platform entitled *Here’s to Tomorrow: Accept Responsibility* which was designed to stress parental responsibility in children’s alcohol initiation [43]. While the policy implications of such activities require further scrutiny, alcohol companies’ CSR tactics have been thriving in framing how personal responsibility associated with alcohol consumption should be understood and communicated.

### CSR as a form of preemptive corporate defense: voluntary regulation

In reviewing the alcohol corporations’ websites and documents, one of the dominant points of their CSR rhetoric is premised on the idea of “voluntarism”. We have observed that the alcohol companies support self-regulation and voluntary market initiatives, which are considered by the public health community as corporate tactics which the alcohol industry uses to preemptively protect business from future regulatory restrictions on their marketing and advertising freedom [44]. The alcohol companies’ promotion of the voluntarist narrative is based on the assumption that the market will effectively resolve many pressing issues surrounding alcohol consumption and proposed solutions should then fit within market mechanisms. Focusing on self-disciplined regulation gives alcohol companies leeway to circumvent and counter unwanted government intrusion into their business profits. As the ICAP’s CSR website comments:

*Government laws and regulations and industry self-regulation can complement each other; some form of co-regulation is becoming the norm around the world. This combination retains an overarching government authority but****helps avoid the unintended consequences of severe restrictions on marketing***[45]*.* [emphasis added]

Similarly, AB InBev stated on its website that its internal code seeks to avoid regulatory repercussions: “By adhering fully to this [voluntary] code, we protect our business from future regulatory restrictions to our current marketing and advertising freedom [46].” The argument that government regulation would hamper voluntary efforts of alcohol corporations to improve their behaviour is illogical but has been presented to further reinforce the ground for corporate voluntarism. ICAP insists that “self-regulation is a flexible instrument, but it can only truly flourish where the legislative framework gives it sufficient scope to do so [47].”

In their bid to oppose any legally binding measures, the three alcohol companies have launched high profile events on self-regulation. One of such efforts was to publicize the training of industry employees and other stakeholders in the alcohol-related industries as proxies. For example, in May 2011, Pernod Ricard launched a *Responsib’ALL Day* programme by rallying the company’s staff members across the regions [48]. In its 2011 annual report, it stated that

***Our employees are front-line ambassadors****. In order to encourage appropriation of this new platform [Responsib’ALL Day* programme*] and especially its adaptation to local contexts, we decided to involve them****through a cascading training programme****. By May 2011, more than 15,000 employees have been trained in CSR*[49]. [emphasis added]

Similarly since 2010, AB InBev launched *Global Be(er) Responsible Day* to encourage its employees to talk to retailers and consumers about responsible drinking [50]. It states that “we support programs that help educate bar and wait staff on how to serve and sell responsibly [51].” In its 2012 press release, the company claimed that it aims to train a total of 1 million people who serve and sell alcohol by the end of 2014 [52]. SABMiller stated on its website that more than 50,000 employees had participated in training about alcohol responsibility and the company’s policies as part of the company’s responsible drinking campaign [53].

The alcohol industry’s voluntarist narrative has also yielded publications of a series of “code of conduct” documents for marketing and advertisements. Voluntary codes of conduct are in-house policies directed to employees in the form of broad statements or more specific but fairly minimal rules, which usually include statements of support for national industry codes of alcohol advertising standards, establishment of internal marketing review committees and systems of self-regulation. Our data shows that guidelines are remarkably similar across different alcohol corporations. For example, SABMiller published *Responsible Drinking Messages in Packaging and Advertising* in April 2012 [54]. Pernod Ricard also issued the *Pernod Ricard Code of Commercial Communications* in June 2012 [55]. Likewise, AB InBev published the *Anheuser-Busch InBev Code of Business Conduct* in August 2011 [56].

All these documents have one commonality: they stress that their advertisements are not targeted at young people below the legal drinking age and that they do not encourage excessive or irresponsible drinking. In contrast to the widely accepted view among public health advocates, the industry has underplayed the impact of alcohol marketing on youths’ decisions to drink: “To our knowledge, studies indicate that advertising has a negligible if any influence on underage drinking [57].” The documents related to the three alcohol companies’ codes of conduct also state that their voluntary codes are externally monitored and regulated. Our closer investigation, however, shows that there is limited independent input and a lack of comprehensive enforcement, leaving important areas under-regulated. For example, SABMiller’s self-regulated packaging and marketing communication materials are assessed by Ebiquity and KPMG, both of which turn out to be private marketing and industry consultant companies while those of Pernod Ricard and AB InBev are monitored by the companies themselves through so-called internal control committees. This suggests that there is little transparency and almost no third party control and sanctions behind the veil of the alcohol companies’ skillfully formulated ethical rules. The sole reliance on self-regulation and lack of public accountability mean that the alcohol industry’s voluntarist narratives are designed to avoid effective alcohol policies.

### CSR as brand marketing and promotion: corporate philanthropy

Corporate philanthropy is another mechanism utilized by alcohol companies as part of their good corporate citizenship activities. The alcohol industry’s engagement in a broad spectrum of philanthropic activities is well described in each alcohol corporation’s website mainly under the theme of sustainable development and humanitarian endeavours.

In reviewing the industry materials, we have found that corporate philanthropy activities of the alcohol companies are characterized by two aspects: social outreach and sponsorship of the arts and cultural events. Social outreach encompasses activities from disaster relief charity work and sponsorships to underserved communities such as hunger and poverty charities to environmental sustainability initiatives including reduction of water use and carbon emission. From the viewpoint of the alcohol industry, corporate philanthropy is not just a strategy for brand promotion but is a tactic to enter, supply and develop local markets. Recent evidence suggests that global alcohol corporations’ philanthropic activities tend to target emerging economies with large youth populations [58]. Fast growing markets for alcohol consumption have become popular destinations for alcohol companies to demonstrate their corporate philanthropy.

A typical example is the *One Rupee Fund* that Pernod Ricard launched for vaccinations and health education in rural India in 2011 [59]. Similarly, AB InBev claimed to have donated USD $38,000 to sponsor children of migrant workers in China in 2010 [60]. Intriguingly, behind their philanthropic activities, one can see the alcohol companies’ intentions for growth in these emerging markets. In its annual report, Pernod Ricard noted that “Growth was remarkable in the two key markets of China and India. China led the way, growing of 14% by volume in the wake of strong demand for ultra-premium brands… India grew 25% [61].” AB InBev also stated that “Our revenue growth was driven primarily by higher volume in Brazil, Argentina and China… Anheuser-Busch InBev has a foot firmly planted in the Chinese market, the largest potential growth pool for the beer industry [62].” On the other end of the spectrum, SABMiller claimed that it pledged USD $34 million in 2012 in community projects primarily focusing on the creation of sustainable economic opportunities for small entrepreneurs in several developing countries [63]. Despite the company’s seemingly altruistic contribution, one can observe a profit-oriented business motive behind its efforts in corporate philanthropy. Statements from Christine Thompson, the policies issues manager of SABMiller plc, indicate that the alcohol company’s small entrepreneur initiatives are not simply driven by benevolent intentions but are also an active attempt to increase corporate domination and opportunity:

*The business objectives for engaging with small-scale farmers have differed from region to region, ranging from strengthening government relations to securing future input supplies… In Uganda and Zambia, smallholder sourcing was central to the launch of a new brand, Eagle Lager, which was only made possible due to special excise reductions agreed with government. In India, the main business driver was to ensure the quality and security of agricultural supply needed to meet rapidly growing demand for SABMiller’s products nationally. In Tanzania, the business benefits were seen as securing the supply of quality barley in the face of uncertain commodity prices and supply, and generating savings of excise tax through import substitution*[64].

The statement above reinforces the criticism that the philanthropic charity work done by the industry has underlying economic motives rather than an altruistic drive towards sustainable development.

Sponsorship of the arts and cultural events is another form of the CSR activities of the alcohol companies. In particular, the wine industry appears to have actively engaged in this type of sponsorship given that wine is claimed to be symbolically associated with culture, art and sophistication. Sponsorship of the arts usually takes place either in the form of endowments and scholarships/awards to young artists or funding to highly publicized cultural events. Pernod Ricard, one of the major players in the global wine market, is seen as an active patron of the arts. In exercising corporate philanthropy, brand name attachment to events was found to be a common corporate tactic. *Havana Cultura*, an annual international cultural event sponsored by Pernod Ricard, illustrates this point [65]. Another industry practice is to exploit popular cultural events as a vehicle to foster advertising and marketing of alcoholic products. Such cultural patronage undoubtedly attracts the attention of the youth population and links alcohol company’s respective brands to the events.

Pernod Ricard states that its sponsorship of daily concerts of the greatest DJs in Thailand held in 2011 was “given full 360° media support with 13,500 new fans on Facebook and 13 million hits on search engines [66].” Similarly, the company’s sponsorship of a youth talent competition in the arts and entertainment in Poland, namely the ABOSULUT initiative, garnered “400,000 visitors” on its website [67]. In a media saturated environment, alcohol companies are seeking to find more innovative ways to promote brand marketing, and CSR offers various effective avenues. Corporate philanthropy is sometimes designed with the intention of promoting alcohol brands. There appears to be an increasingly high level of expenditure worldwide on sponsorship as an important form of CSR.

## Discussion

Our analysis identified three CSR tactics employed by the alcohol corporations which are closely tied in with the industry’s underlying corporate intents. First, alcohol manufacturers employ CSR to frame issues, define problems and guide policy debates. For example, by seizing the initiative to discuss the genesis of alcohol-related problems, the industry is able to pinpoint whom or what is to be blamed. The CSR narratives hardly locate any responsibility with the industry itself [68]. Instead, the alcohol industry promotes an idea that alcohol can cause problems only in the hands of a small number of users – who are by nature abusive and reckless (consequently negligent) – and therefore this small group is to be blamed. Arguably, this “personal responsibility” narrative manifested in the alcohol companies’ CSR is designed to shield the alcohol industry from mounting criticism of aggressive marketing practices that normalize alcohol drinking as part of everyday life. Findings from our study evidently indicate that the alcohol corporations do not support the implementation of any restrictive measures on the alcohol industry’s behaviour or policies that aim at the reduction of the overall volume of alcohol consumption [69,70]. There is also evidence that educational interventions, heavily reliant on the idea of “personal responsibility”, do not reduce alcohol consumption or alcohol related-harm [71,72]. The personal responsibility frame put forward by the alcohol industry emphasises individual failings, effectively shifting the attention from those who manufacture and promote the products to those who consume them.

Second, the alcohol manufacturers’ CSR initiative promotes voluntary regulation to limit the purview of governmental regulation [73,74]. Underscoring the argument for self-regulation is the rosy view that members of alcohol industry are better-suited to monitoring rule-following rather than through mandatory legal regulation. Our findings indicate however that the industry’s CSR accounts seriously lack transparency and public accountability because the alcohol manufactures’ self-regulatory practices have been rarely monitored and assessed by independent bodies. This is in keeping with the existing analyses that industry self-regulatory codes are ineffective and highly subjective [75-77]. The very fact that the alcohol industry has an apparent aversion to legislative restrictions directly contradicts its claims that the industry as a whole can be a good corporate citizen. There is a fundamental and irreconcilable conflict of interest here. The alcohol industry’s claim on self-regulation has dire policy implications as it usurps the government’s responsibility to provide the duty of care to the populace, and devolves the responsibility to the hands of a few private entities who are accountable only to shareholders [78].

The alcohol industry’s third mainstream CSR tactic is philanthropic sponsorship for development issues and cultural events. Contrary to the righteous goal of corporate philanthropy portrayed by the companies, we have observed that the alcohol corporations undertook philanthropic sponsorships for two main reasons. First, it is a means of indirect brand marketing. Second, it allows the alcohol industry to gain preferential access to emerging alcohol markets. One of our findings reveals that “brand stretching” (advertising of non-alcohol events carrying alcohol brand names) is a prominent feature in the industry’s supposed benevolent activities [79]. Research suggests that the impact of corporate philanthropy goes much deeper than traditional marketing strategy [80]. Since the philanthropic sponsorship is conducted under the CSR activities, it is difficult for local governments and international bodies to monitor and regulate these activities. As such, the alcohol industry’s CSR philanthropy can pose a major challenge for public health policy. The absence of clear guidelines to address the adverse effects of corporate philanthropy on public health gives rise to further difficulties.

In light of the evidence found in our paper, several contradictions in the alcohol industry’s CSR activities are apparent. Should a corporation whose main concern is with yielding profit (and thus selling more alcoholic beverages) be permitted to participate in campaigns to regulate the promotion, sale and consumption of alcohol? Would an approach in favour of the personal responsibility of individual drinkers be sufficient to prevent alcohol-related harm without active enforcement of regulations? How could we conceive of the alcohol industry’s philanthropic sponsorships in the absence of a clear monitoring of its business intent? Would a public health strategy aimed at reducing overall consumption of alcohol be compatible with the industry’s strategy, which is to promote overall consumption albeit responsibly? [81] Should a company that sponsors popular events and targets the youth be allowed to contribute to programs that are designed to prevent the very same youth from drinking? The core of these contradictions is that there is a conflict of interest for the alcohol industry when it comes to mitigating the “negatives” on which their business relies. The alcohol industry’s CSR efforts as a whole can never eradicate the “negatives” - at best, the CSR efforts can conceal them but at worst, it would accentuate them. In essence, the alcohol industry’s CSR claims directly contradict any illusion of improved corporate behaviour.

A recent trend indicates that alcohol industry’s CSR efforts have been extended to working in partnership with governments in public health policy making. Through a co-regulation framework, the industry members have increasingly engaged in formulating policy agendas and undertaking the delivery of policy solutions. The advent of industry partnerships is well exemplified by the UK Public Health Responsibility Deal [82] where alcohol corporations and business organizations are invited to join a partnership with government to curb alcohol misuse and its associated social harms. Similar experiences can be found in the European Alcohol and Health Forum [83], the Implementation of Partnership Agreement in Scotland [84], and various alcohol policy initiatives in countries of sub-Saharan Africa [85]. Involvement of alcohol corporations as partners in the policy making was claimed to have yielded certain public health policy steps. For instance, the legislation of the minimum unit pricing of alcohol in Scotland is seen as a small step forward. However, there is little evidence that a concerted action to tackle the increasingly innovative marketing of alcohol products is under way through such public-private partnership [86,87]. It remains to be seen if this could be a “one step forward, two steps backwards” situation.

The public health community has cautioned that the strategy and influence of these corporations can steer discussion away from effective alcohol control measures without many realizing it [88]. Indeed, the alcohol corporations seem to have been successful in framing the nature of alcohol problems by generating distorted views of evidence, thereby shaping policy responses in particular ways [89-91]. Research suggests that policy outcomes emerged from recent evolution of CSR partnerships are consonant with the view of alcohol industry, an approach that highlights “irresponsible drinking” and eschews regulation of the industry’s corporate practices [92,93]. This means that the role of industry CSR in public health policy needs to be subjected to closer scrutiny and that conflict of interest should be rigorously managed in any partnership working with alcohol corporations.

The observations on the alcohol industry’s CSR activities remind us of the political tactics employed by other corporate sectors amidst growing pressures from the public regarding the societal ills they produced [94,95]. For example, the tobacco industry employed political and public relations strategies to effectively thwart the much needed changes in countries where robust control measures were generally absent. That such strategies are appealing for the alcohol industry is not unforeseen and to a large extent, we see similarities in the behaviour of actors in the tobacco and alcohol industries [96]. What is noteworthy is that the alcohol industry is more than ever expanding its CSR strategies to further diminish and inverse its responsibility for alcohol-related problems across both the developed and more worryingly the developing world at an alarming rate with no sign of abatement.

## Conclusions

This paper has demonstrated that the alcohol industry employs CSR as a means to appear as a responsible actor while shaping and reinforcing the industry’s firmly established positions on key alcohol policy issues. Policy officials should remain wary of the true purpose and scope of activities portrayed as socially responsible by the alcohol industry. Public health advocates should closely monitor, scrutinize, and challenge as necessary every altruistic claim that the alcohol industry makes [97]. What is also essential is for scholars and government officials to critically assess the alcohol industry’s involvement in government and policy initiatives that alters what might otherwise have been depicted as a profit-driven economic entity into a palatable public health advocate. More fundamentally, the implementation of any measures on alcohol control should include banning or restricting the publicity efforts of the industry’s CSR [98]. This may include but not limited to mandatory disclosure of conflicts of interest in any industry sponsorship or in-kind contribution, prohibition on CSR advertising that directly or indirectly promotes alcohol brands and products, and compliance inspections and penalties through enforceable legal measures.

It is, however, doubtful that mandated action would be introduced at the national level in the absence of supporting collective efforts and global health governance particularly given the sheer scale and magnitude of the political influence of transnational alcohol companies [99-101]. Lessons are learned in the tobacco arena when an international convention - Framework Convention on Tobacco Control (FCTC) - has made remarkable global impacts in providing an impetus to local and national tobacco control initiatives [102,103]. FCTC recognizes that the tobacco industry employs CSR to undermine and weaken tobacco control policies. More specifically, Article 5.3 of the Convention and its Guidelines demand the commitment of all Parties to protect public health polices from commercial and other vested interests of the tobacco industry [104,105]. A robust body of research into industry documents and case studies further provides an insight into a wide array of measures in response the tobacco industry’s CSR tactics that are increasingly more sophisticated and diverse [106-112]. As a result, the past few years have seen a strengthening of measures curbing industry CSR activities with countries moving from weak policies to more comprehensive restrictions [113].

The remarkable achievement and the breadth of international collaboration in tobacco control through FCTC provide an encouraging instance of public health being able to counter corporate vested interests. It is clear that a similar governance mechanism could be developed to support coordinated alcohol control efforts. Our findings underline the importance of developing an equivalent international instrument for alcohol control globally. It is only through such internationally binding measures that more commitments and resources to advancing alcohol control can be achieved.

## Abbreviations

AB InBev: Anheuser-Busch InBev; CSR: Corporate social responsibility; FCTC: Framework convention on tobacco control; ICAP: International centre for alcohol policies

## Competing interests

The authors declare that they have no competing interests.

## Authors’ contributions

SY and THL performed the analysis. SY drafted the manuscript. THL revised the manuscript. Both authors read and approved the final manuscript.

## Pre-publication history

The pre-publication history for this paper can be accessed here:

http://www.biomedcentral.com/1471-2458/13/630/prepub
